# Notch Signaling Inhibition Alleviates Allergies Caused by Antarctic Krill Tropomyosin through Improving Th1/Th2 Imbalance and Modulating Gut Microbiota

**DOI:** 10.3390/foods13081144

**Published:** 2024-04-09

**Authors:** Na Lin, Hai Chi, Quanyou Guo, Zhidong Liu, Ling Ni

**Affiliations:** 1 East China Sea Fisheries Research Institute, Chinese Academy of Fishery Sciences, Shanghai 200090, China; lina903368043@163.com (N.L.); dhsguoqy@163.com (Q.G.); ni.lu.yi2006@163.com (L.N.); 2 College of Food Science and Engineering, Dalian Ocean University, Dalian 116023, China

**Keywords:** antarctic krill tropomyosin, food allergy, Notch signaling, Th1/Th2 imbalance, gut microbiota, SCFAs

## Abstract

Antarctic krill tropomyosin (AkTM) has been shown in mice to cause IgE-mediated food allergy. The objective of this work was to investigate the role of Notch signaling in AkTM-sensitized mice, as well as to determine the changes in gut microbiota composition and short-chain fatty acids (SCFAs) in the allergic mice. An AkTM-induced food allergy mouse model was built and *N*-[*N*-(3,5-difluorophenacetyl)-*L*-alanyl]-*S*-phenylglycine t-butyl ester (DAPT) was used as an γ-secretase inhibitor to inhibit the activation of Notch signaling. Food allergy indices, some key transcription factors, histologic alterations in the small intestine, and changes in gut microbiota composition were examined. The results showed that DAPT inhibited Notch signaling, which reduced AkTM-specific IgE, suppressed mast cell degranulation, decreased IL-4 but increased IFN-γ production, and alleviated allergic symptoms. Quantitative real-time PCR and Western blotting analyses revealed that expressions of Hes-1, Gata3, and IL-4 were down-regulated after DAPT treatment, accompanied by increases in T-bet and IFN-γ, indicating that Notch signaling was active in AkTM-sensitized mice and blocking it could reverse the Th1/Th2 imbalance. Expressions of key transcription factors revealed that Notch signaling could promote Th2 cell differentiation in sensitized mice. Furthermore, 16S rRNA sequencing results revealed that AkTM could alter the diversity and composition of gut microbiota in mice, leading to increases in inflammation-inducing bacteria such as *Enterococcus* and *Escherichia-Shigella*. Correlation analysis indicated that reduced SCFA concentrations in AkTM-allergic mice may be related to decreases in certain SCFA-producing bacteria, such as *Clostridia_UCG-014*. The changes in gut microbiota and SCFAs could be partially restored by DAPT treatment. Our findings showed that inhibiting Notch signaling could alleviate AkTM-induced food allergy by correcting Th1/Th2 imbalance and modulating the gut microbiota.

## 1. Introduction

Antarctic krill (*Euphausia superba*) has the potential to be developed as numerous high-value-added products for the human diet and be a source of high-quality protein and high nutritional content with vast biological resources [1]. The potential allergenicity of Antarctic krill has gained considerable attention due to its widespread usage as a culinary raw material. Tropomyosin (TM) has been identified as the main allergen in Antarctic krill by researchers [2,3]. Lin et al. demonstrated that Antarctic krill TM (AkTM) has a high sensitization capacity and can cause IgE-mediated allergic responses in TM-sensitized mice [2].

Food allergy is an immunological response to specific dietary components that is damaging to human health [4]. Crustaceans, such as shrimps and crabs, fall under one of the eight categories of food allergens, producing allergic reactions in many consumers. Food allergies, particularly those caused by crustacean aquatic items, are mostly mediated by IgE [5]. IgE-mediated food allergy can be split into sensitization, eliciting, and effector phases. Initially, antigen presenting cells (APCs) processed the antigen and delivered it to the immune cell. The naïve CD4^+^ T cells differentiate into Th2 cells with the aid of co-stimulation signals and cytokines. Subsequently, B cells were activated and became IgE- and IgG1-producing plasma cells under the stimulation of IL-4, IL-5, and IL-13. Antigen-specific IgE that is newly generated binds to FcεRI receptors on mast cells/basophils, leading the body to become sensitized. When exposed to the same antigen, sIgE would cross-link with FcεRI on the surface of effector cells, resulting in the release of allergic mediators such as histamine, proteases, and so on. The released inflammatory mediators act on target organs, causing an immediate allergic reaction [5,6]. As a result, food allergy is a tightly controlled immunological process involving a variety of immune cells, cytokines, and immune components that engage in numerous signal pathways. Complex molecular mechanisms and cellular processes are included in these pathways. The potential sensitization mechanism of AkTM was investigated in this work using a TM-sensitized mouse model.

Some evidence shows that Notch signaling is important in T cell-mediated immunological responses, notably in the modulation of Th cell differentiation [7,8]. Notch signaling has been demonstrated to play a critical role in IgE-mediated food allergy through facilitating Th2 polarization and enhancing mast cell degranulation and Th2 cytokine production [9,10,11]. Notch signaling is very conserved, with four receptors (Notch1–4) and five ligands (Dll1, Dll3, Dll4, Jag1, Jag2). The ligand–receptor contact initiates Notch signaling, followed by two proteolytic cleavages. The last cleavage results in the release of the Notch intracellular domain (NICD), which is mediated by γ-secretase activity. The freed NICD then translocates to the nucleus and binds to the transcription factor CSL, therefore initiating the transcription of target genes [12]. Notch signaling can control cell differentiation, fate choices, and proliferation. What ligands, for example, are utilized to activate this signaling pathway, which may alter the cell fate decision between Th1 and Th2 [7]? Notch signaling regulates the production of the Gata3 gene, a transcription factor required for Th2 cell differentiation [13]. Although Notch plays a role in promoting Th1 cell lineage, it is not required for Th1 cell development [7,8,14]. Many studies have demonstrated that inhibiting Notch signaling with a γ-secretase inhibitor (GSI) can limit Th2 polarization, which might be a potential method for treating Th2-mediated diseases such as allergic asthma and food allergies [10,11,15]. *N*-[*N*-(3,5-difluorophenacetyl)-*L*-alanyl]-*S*-phenylglycine t-butyl ester (DAPT) inhibits the γ-secretase, preventing abnormal amyloid precursor protein cleavage. It is also used to inhibit the activation of Notch signaling [16]. In this research, we explored the roles of Notch signaling by DAPT treatment in the allergy caused by AkTM.

Emerging evidence suggests that gut microbiota and their metabolites, short-chain fatty acids (SCFAs), play an important role in the development of IgE-mediated food allergy [17,18,19]. Numerous studies have found that the gut microbiota play a significant role in the intestinal mucosal immune system. The dysbiosis of gut microbiota disrupts the intestinal barrier, increasing antigen access, and it alters host–microbiota equilibrium, disrupting the balance of Th1/Th2 and triggering allergic and inflammatory reactions [20]. SCFAs, such as acetate, propionate, and butyrate, are dietary fiber fermentation products produced by the gut microbiota, and they can effectively modulate the mucosal immune system, responding by protecting against food allergy [21,22]. Acetate, for instance, can promote intestinal IgA responses via the GPR43 receptor [23]. Propionate treatment in mice increased the development of DC precursors and macrophages via receptor GPR41, which suppressed Th2 cells, reducing allergic inflammation [24]. Taken together, SCFAs can modulate food allergy by activating GPRs and increasing the integrity of the intestinal epithelium barrier.

In this study, we explored the roles of Notch signaling in the allergy caused by AkTM using a TM-sensitized mice model. DAPT was used as a GSI to suppress the Notch signaling pathway. Allergy-related indices as well as signaling molecules involved in the pathway were examined. Moreover, the composition and diversity of the gut microbiota, as well as their relations with SCFAs, were studied in mice from the AkTM, DAPT, and normal control groups. We proposed the hypothesis that Notch signaling was active in IgE-mediated food allergy and that inhibiting Notch signaling might affect allergic reactions in AkTM-sensitized mice.

## 2. Materials and Methods

### 2.1. Antarctic Krill TM

AkTM was extracted and purified as our previous work described [2]. We have demonstrated that AkTM has a strong sensitization capacity and can provoke IgE-mediated allergic reactions in mice [2].

### 2.2. Protocols of Animal Experiment

Female BALB/c mice (five weeks old and 17~19 g weight), free of specific pathogens, were maintained at 23 ± 1 °C and 50 ± 5% humidity with a 12-h light/dark cycle. Mice were acclimatized for 1 week before conducting the sensitization experiment. All animal experiments were carried out in compliance with guidelines approved by the Animal Ethical and Welfare Committee of Pony Testing International Group Co., Ltd (Beijing, China).

BALB/c mice were randomly divided into three groups (*n* = 10, per group): the normal control (NC) group; the model control (MC) group, AkTM + 1% DMSO; and the DAPT group, AkTM + DAPT (5 mg/kg) [9,11]. Mice were given 200 μg/mouse AkTM intraperitoneally on days 0, 7, and 14, and then challenged with 600 μg/mouse AkTM on days 21, 28, and 35. AkTM was dissolved in 100 μL of PBS and then mixed with 100 μL of Freund’s adjuvant. In the DAPT group, DAPT dissolved in 1% DMSO was administrated intraperitoneally 1 h before each AkTM sensitization or challenge. The NC group was injected with 200 μL of PBS. In addition, 1% DMSO served as a control for DAPT in the MC group. Blood samples were drawn from the orbital plexus on days 27 and 35, followed by centrifugation using 3000× *g* at 4 °C for 10 min to obtain the serum. All mice were sedated and killed 24 hours after the final challenge, and the spleens, small intestine, and intestinal contents were collected for future use. The protocol for the AkTM-sensitized and DAPT-treated mice model is shown in Figure S1. Anaphylactic symptoms of mice were evaluated 30 min after the last challenge using the scoring criteria described by Pablos-Tanarro et al. [25].

### 2.3. Spleen Cells Culture and Cytokine Assay

Spleens were obtained from each group of mice and processed into a single-cell suspension using the procedure described in our previous work [2]. Subsequently, the cultivated cells were divided into three groups, the control group, DMSO group, and DAPT group, which were incubated with culture medium, 0.1% DMSO, and 20 μM DAPT, respectively. After an incubation of 2 h, all cells were stimulated with AkTM at a final concentration of 0.5 μg/μL. After incubation and centrifugation, the cell supernatants were collected and used to detect cytokines of IL-4 and IFN-γ using commercial ELISA kits (Abmart, Shanghai, China).

### 2.4. Measurement of Serum Antibodies, Histamine and mMCP-1

Serum AkTM-specific IgE and specific IgG1 were measured by indirect ELISA as our work previously described [2].

Levels of serum histamine and mMCP-1 were measured using mouse ELISA kits (Abmart, Shanghai, China) according to the manufacturer’s instructions.

### 2.5. Quantitative Real-Time PCR (qPCR)

Total RNA was extracted from spleen tissues using an RNAeasy kit (Beyotime, Shanghai, China) according to the manufacturer’s instructions, and cDNA was produced by reverse transcription with SuperScript IV (reverse transcriptase, Invitrogen, Carlsbad, CA, USA) from the total RNA. The expression of mRNA was measured by qPCR using QuantStudio™ 6 Flex (Applied Biosystems, Carlsbad, CA, USA) with the SYBR Green qPCR Mix kit (Beyotime, Shanghai, China). GAPDH was used as the internal control. Sequences of the primer are listed in Table S1. The relative expression levels of mRNA were calculated by the 2^−ΔΔCt^ method using GAPDH mRNA for normalization.

### 2.6. Western Blotting

Spleen tissues were ground into a powder and lysed in RIPA with a PMSF protease inhibitor. After 1 h in an ice bath, the mixtures were centrifugated and the supernatants were collected for SDS-PAGE analysis. Next, 30 μg proteins of each sample were loaded into the SDS-PAGE gels, and then the proteins were transferred to NC membranes under low temperatures. Subsequently, the NC membranes were blocked with 5% nonfat milk powder, followed by probing with primary antibodies against Gata3, T-bet, Hes-1, IL-4Rα, NF-κB, p-NF-κB, and β-actin overnight at 4 °C. After washing, the membranes were treated with HRP-conjugated anti-mouse or anti-rabbit IgG antibodies (Proteintech). The membranes were then stained with ECL reagents and exposed to film. For the purpose of semi-quantification, the gray values of blots were analyzed by ImageJ software (ImageJ 1.52a).

### 2.7. Histologic Staining

The jejunum tissues were collected from mice, fixed in 4% paraformaldehyde, and then embedded in paraffin. Five-micrometer-thickness sections were dyed with hematoxylin and eosin (HE) for the examination of histologic alterations in the small intestine. Toluidine blue staining was performed to assess mast cell degranulation in the jejunum. The process of toluidine blue staining was as follows: tissues were dewaxed to water, dyed with toluidine blue working solution, washed with water, dehydrated using 95% ethanol and absolute ethanol, and then made transparent with xylene and sealed with resinene.

### 2.8. 16S rRNA Gene Sequencing and Data Analysis

DNA extraction (from colon contents) and 16S rRNA gene sequencing were performed using the method described by Zhou et al. [26].

The raw gene sequencing reads were processed as described by Zhou et al. [26]. Statistical analyses of gut microbiota were performed using the online Majorbio Cloud platform https://cloud.majorbio.com (accessed on 10 October 2023). Alpha indices of Ace, Chao, Shannon, and Sobs analyses were performed using R-3.3.1 software. The *p* value and FDR value (*p*-*adjust* value) were used for comparing the differences between groups. Hierarchical clustering, PCoA, and NMDS analyses of beta–diversity based on Bray–Curtis dissimilarity or weighted unifrac distances were carried out by the Vegan v2.5-3 package. A linear discriminant analysis (LDA) was used to identify the significantly differential abundant taxa of microbiota among different groups (LDA score > 4, and *p* < 0.05).

### 2.9. SCFAs Extraction and Analysis

The colon contents (20 mg) of mice were mixed with 800 μL of 0.5% phosphoric acid solution containing an internal standard 2-ethylbutyric acid to a final concentration of 10 μg/mL. The mixture was ground, sonicated, and centrifuged. Subsequently, the supernatant was obtained and mixed with 200 μL *n*-butanol, followed by vortex, ultrasound, and centrifugation. The supernatant was collected and placed in a vial for GC-MS analysis. A standard mixture solution of acetate, propionate, butyrate, isobutyrate, and hexanoate was prepared using their stock solutions, respectively. SCFA contents in samples were calculated through the standard curve.

The analytical instrument was the 8890B-7000D GC/MSD system (Agilent Technologies, Santa Clara, CA, USA). An HP FFAP capillary column (30 m × 0.25 mm × 0.25 μm) was used to separate the SCFAs, and the flow rate of helium was 1 mL/min. The injector temperature was 180 °C, and the samples were injected in a split mode (10:1) in a volume of 1 μL. The initial oven temperature was 80 °C, raised to 120 °C at a speed of 20 °C/min, then raised to 160 °C at 5 °C/min, and finally held at 220 °C for 3 min. The ion source of the mass detector was EI and its temperature was set as 230 °C. The electron collision energy was 70 eV and samples were detected by a SIM mode. Data acquisition and processing were performed using a Masshunter software (v10.0.707.0).

### 2.10. Statistical Analysis

All the quantitative data was presented as means ± standard deviation (SD). Data differences among different groups were analyzed using the one-way ANOVA method (IBM SPSS 22) with *p* < 0.05 or *p* < 0.01 considered as statistically significant. Correlation analysis between SCFA contents or the allergic indices, and the abundances of microbiota, was performed using Spearman’s analysis.

## 3. Results

### 3.1. DAPT Treatment Alleviated Allergic Symptoms, Reduced the Levels of Specific IgE, and Affected the Secretion of Cytokines

**Figure 1:**
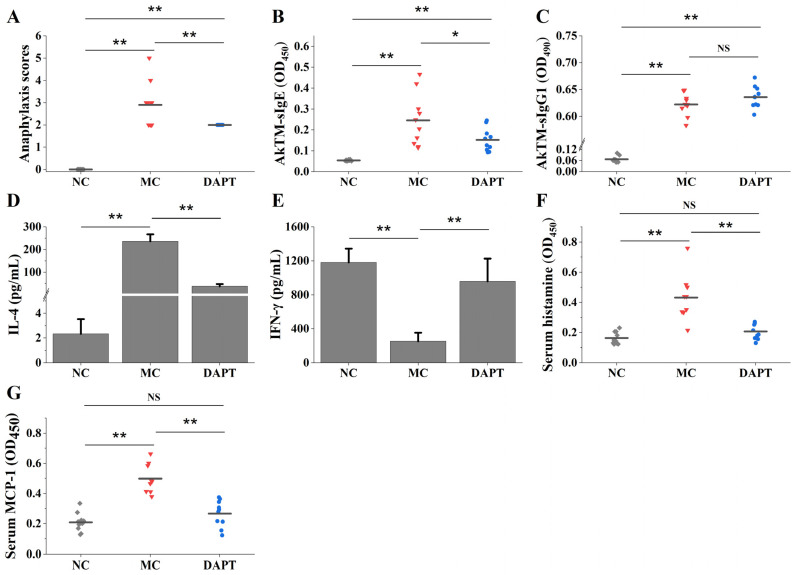
Anaphylaxis scores and allergic indices in mice of different groups. (**A**) Anaphylaxis scores; levels of serum (**B**) AkTM-specific IgE and (**C**) AkTM-specific IgG1 (*n* = 10); levels of cytokines (**D**) IL-4 and (**E**) IFN-γ (*n* = 4); levels of serum (**F**) histamine and (**G**) mMCP-1 (*n* = 10). * *p* < 0.05, ** *p* < 0.01, and NS means no significant differences.

An AkTM-sensitized BALB/c mouse model [2] was used to assess the influence of DAPT treatment on the anaphylactic reaction. Figure 1A shows that AkTM-sensitized mice exhibited greater anaphylaxis scores (*p* < 0.01, vs. NC group). Compared with the MC group, the DAPT group showed less allergic symptoms (*p* < 0.01), suggesting that DAPT treatment could alleviate allergic symptoms caused by AkTM.

Significantly higher levels of AkTM-specific IgE and IgG1 were observed in the MC group (Figure 1B,C, *p* < 0.01, vs. NC group), indicating that an effective allergy model had been constructed. Compared with the MC group, levels of serum-specific IgE in the DAPT group were significantly reduced (*p* < 0.05), indicating that the IgE antibody response was considerably inhibited following DAPT treatment. It is noteworthy that the specific IgG1 in the DAPT group had no significant difference when compared with the MC group (Figure 1C, *p* > 0.05).

To investigate the impact of DAPT on Th1 and Th2 cytokine secretion, splenocytes from mice in each group were co-cultured with or without DAPT before being stimulated with the AkTM antigen. The IL-4 level increased dramatically in splenocytes from the MC and DAPT group (Figure 1D, *p* < 0.01, vs. NC group). IL-4 levels in the DAPT group were considerably lower than in the MC group (*p* < 0.01). The Th1 cytokine of IFN-γ was strikingly decreased in the MC group (*p* < 0.01, vs. NC group), but significantly increased after DAPT treatment (Figure 1E, *p* < 0.01, DAPT vs. MC group). These findings suggested that DAPT treatment could inhibit Th2 cytokine production while promoting Th1 cytokine production, suggesting a shift from the Th2 response to the Th1 response.

### 3.2. DAPT Treatment Reduced Levels of Serum Histamine and mMCP-1

Histamine and mMCP-1 are two important indicators of mast cell degranulation [27], so the two indices were measured to evaluate the effect of DAPT on cell degranulation. As shown in Figure 1F,G, serum histamine and mMCP-1 levels were remarkably increased in the MC group (*p* < 0.01, vs. NC group). The DAPT group, on the other hand, demonstrated a significant decrease in both indices (*p* < 0.01, vs. MC group). These findings suggest that mast cell degranulation occurred in the AkTM-allergic mice and that DAPT treatment could suppress mast cell degranulation in allergic mice.

### 3.3. Effects of DAPT Treatment on the Expression of Hes-1, Gata3 and T-Bet

**Figure 2:**
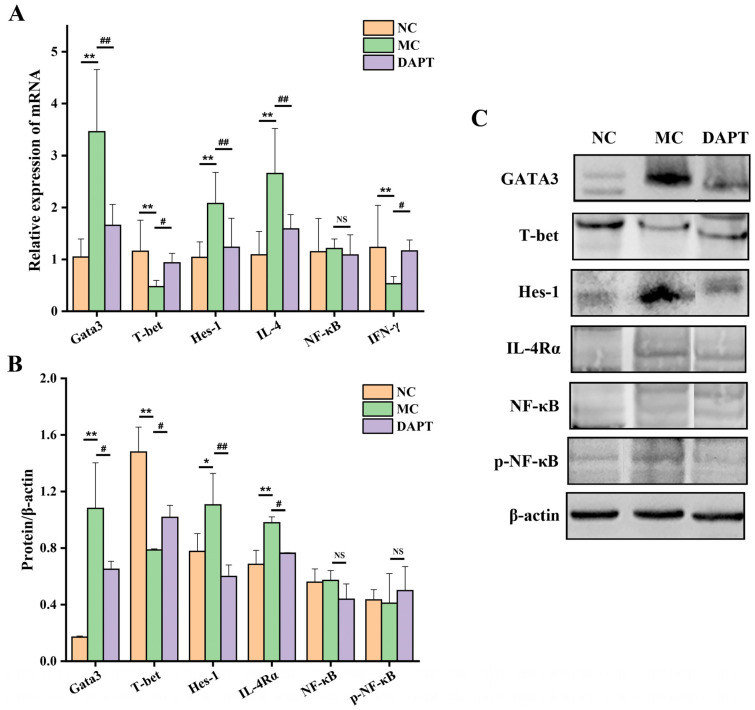
mRNA and protein expressions of Notch signaling and Th cell differentiation related factors in different groups (*n* = 3). (**A**) mRNA expressions of T-bet, Gata3, Hes-1, IL-4, IFN-γ, and NF-κB; (**B**) protein expressions of Gata3, T-bet, Hes-1, IL-4Rα, NF-κB, and p-NF-κB; (**C**) Western blotting analysis of proteins. β-actin, an internal reference protein. * *p* < 0.05, ** *p* < 0.01, vs. NC group; # *p* < 0.05, ## *p* < 0.01, vs. MC group, and NS means no significant differences.

The expression of Hes-1, a direct transcriptional target gene of Notch signaling [28], was detected at the mRNA and protein levels to evaluate the influence of DAPT on Notch signaling. Mice in the MC group showed enhanced Hes-1 mRNA expression (Figure 2A, *p* < 0.01, vs. NC group). Consistent with the mRNA level, Hes-1 protein expression was up-regulated in the MC group (Figure 2B,C, *p* < 0.05, vs. NC group). The results suggested that Notch signaling was involved in the development of food allergies induced by AkTM. DAPT treatment reduced Hes-1 expression significantly at the mRNA and protein levels (Figure 2, *p* < 0.01, vs. MC group), indicating that DAPT effectively inhibited Notch signaling in allergic mice. The effects of DAPT on the expression of Gata3 and T-bet, which are critical transcription factors required for the differentiation of Th2 and Th1 cells [29,30,31], respectively, were examined by qPCR and Western blotting. Both mRNA and protein levels of Gata3 were remarkably increased in the MC group, whereas the T-bet expression levels were significantly decreased in this group (Figure 2, *p* < 0.01, vs. NC group). The results demonstrated that a Th2 polarization occurred in the AkTM-sensitized mice. After administration with DAPT, the sensitized mice showed decreased Gata3 expression at both the mRNA and protein levels (*p* < 0.01 or 0.05), whereas the T-bet expression was significantly increased in the DAPT group (Figure 2, *p* < 0.05, vs. MC group). These findings indicated that the inhibition of Notch signaling by DAPT could down-regulate Gata3 expression while up-regulating T-bet expression. 

### 3.4. Effects of DAPT Treatment on the mRNA Levels of IL-4 and IFN-γ

The mRNA expressions of Th2 cytokine IL-4 and Th1 cytokine IFN-γ were also determined to evaluate the effect of DAPT on the cytokine production of immune cells. As shown in Figure 2A, the level of IL-4 mRNA in AkTM-sensitized mice was increased (*p* < 0.01, vs. NC group), while the IFN-γ mRNA expression was decreased in the MC group (*p* < 0.01). After DAPT treatment, the mRNA expression of IL-4 was significantly decreased (*p* < 0.01), whereas the IFN-γ mRNA level was increased (*p* < 0.05, vs. MC group). The mRNA expressions of IL-4 and IFN-γ in spleens were consistent with the results obtained by detecting cytokine levels in the supernatant of splenocytes (Figure 1D,E). Combined with the expression levels of Gata3 and T-bet, we can conclude that Th1/Th2 imbalance occurred in AkTM-sensitized mice and DAPT treatment could improve the imbalance by inhibiting the Th2 response and promoting Th1 response.

### 3.5. Effects of DAPT Treatment on the Expression of IL-4Rα, NF-κB and p-NF-κB

IL-4Rα is an IL-4 receptor on naïve CD4 cells that can be activated by IL-4 and promote the subsequent signal transduction in the canonical differentiation pathway of Th2 cells [6]. The expression of IL-4Rα was analyzed by Western blotting to assess the effect of DAPT on it. As shown in Figure 2B,C (Figures S4–S9) the expression of IL-4Rα was significantly increased in the MC group (*p* < 0.01, vs. NC group), while DAPT treatment effectively reduced its expression (*p* < 0.05, vs. MC group). The expression of NF-κB, which is considered to play an important role in the immune process and in inflammatory diseases [32], was also measured in this study. NF-κB and p-NF-κB protein expression levels, as well as the NF-κB mRNA level, showed no significant differences among the three groups (Figure 2). Although NF-κB was related to the production of Th2 cytokines in Th2 cell differentiation [32], we did not observe any significant changes in the expression level of this transcription factor. The findings suggest that NF-κB might not be involved in Th2 cell differentiation in AkTM-sensitized mice, and DAPT had no effect on NF-κB or p-NF-κB expression under this circumstance.

### 3.6. Histopathological Examination of Small Intestine

**Figure 3:**
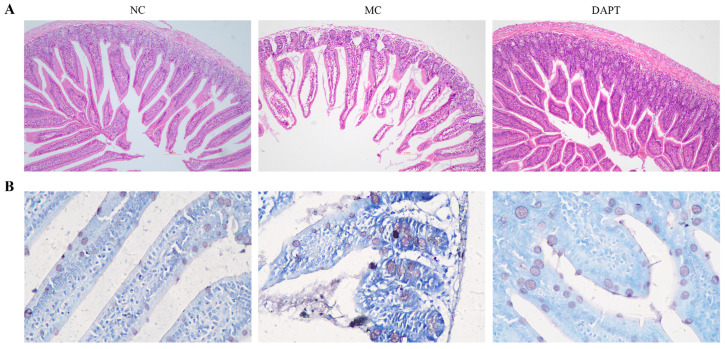
Histologic staining of jejunum tissues of mice. (**A**) Hematoxylin and eosin staining (×100); (**B**) toluidine blue staining (×400).

To evaluate the impact of AkTM sensitization and DAPT treatment on the intestinal mucosa in mice, HE staining and a histopathological analysis of jejunum tissues were performed. As shown in Figure 3A, the intestinal mucosa of mice in the NC group presented neatly arranged intestinal villi and epithelial cells. The morphology and structure of the small intestine were normal, and no evident inflammation occurred. In the MC group, accompanied by symptoms of diarrhea in mice, the epithelial cells at the tip of the intestinal villi sloughed and muscular layer width decreased. Compared with the NC group, the intestinal mucosa in the MC group was damaged and showed a significant infiltration of inflammatory cells. Mice in the DAPT group showed normal small intestine morphology and no evident inflammatory phenomena. The results indicated that the inhibition of Notch signaling by DAPT could effectively alleviate allergic symptoms in AkTM-allergic mice and also protect the integrity of the intestinal mucosa. In the NC group, toluidine blue staining of the jejunum displayed clearly outlined mast cells with intact cell membranes (Figure 3B). However, in the MC group, the number of intestinal mast cells increased, and most mast cell membranes ruptured, with blurred outlines and degranulation reactions (Figure 3B). The integrity, clear outlines, and reduced numbers of mast cells in the DAPT group (vs. MC group) indicated that mast cell degranulation was greatly suppressed after DAPT inhibiting Notch signaling. The results were consistent with the detection results of histamine and mMCP-1 secretion levels (Figure 1).

### 3.7. Gut microbiota Diversity Analysis

**Figure 4:**
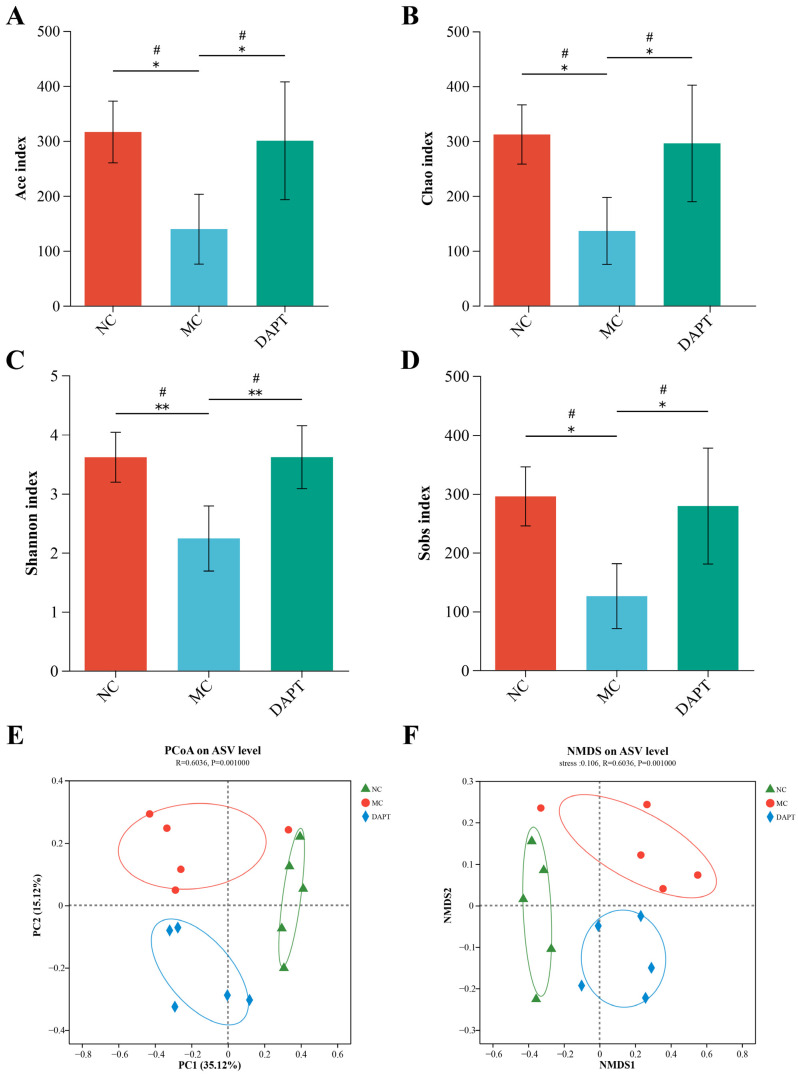
The diversity analysis of gut microbiota from mice (*n* = 5). Alpha−diversity indices were assessed by (**A**) Ace, (**B**) Chao, (**C**) Shannon, and (**D**) Sobs; beta−diversity was analyzed by (**E**) PCoA and (**F**) NMDS based on Bray–Curtis distances. * *p* < 0.05, ** *p* < 0.01; # *p*-*adjust* < 0.05.

To investigate the microbial community changes among different treatment groups, 16S rRNA gene sequencing of intestinal microbiota was conducted. The alpha−diversity indices of Ace, Chao, and Sobs analyses showed that the community richness of the MC group was remarkably reduced compared to the NC group (*p* < 0.05 and *p*-*adjust* < 0.05, Figure 4A,B,D). Similarly, the three alpha indices of the DAPT group were higher than those of the MC group. The Shannon index, which reflects microbial community diversity, was significantly lower in the MC group than in the other two groups (*p* < 0.01 and *p*-*adjust* < 0.05, Figure 4C). Beta−diversity was assessed by hierarchical clustering, PCoA, and NMDS analyses. PCoA and NMDS analyses using Bray–Curtis dissimilarity revealed significant differences across the three groups (*p* = 0.001, Figure 4E,F), indicating changes in the gut microbiota of mice. In addition, PCoA based on weighted unifrac, and hierarchical clustering based on Bray–Curtis distance analyses obtained similar results to those above (Figure S2).

### 3.8. Changes in the Gut Microbiota Composition at Different Taxonomic Levels

**Figure 5:**
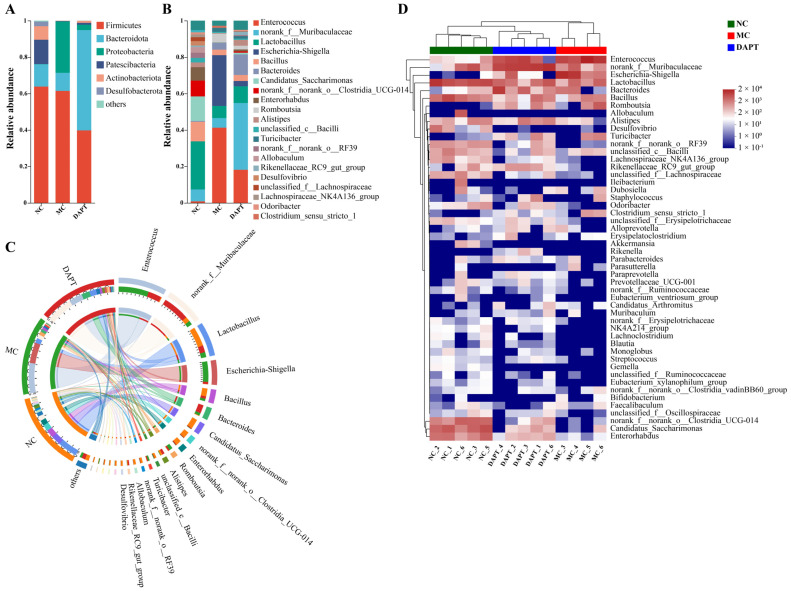
Gut microbial composition of mice (*n* = 5 for NC and DAPT groups; *n* = 4 for MC group). Bar−plot analysis of microbiota composition at the phylum (**A**) and genus (**B**) levels; Circos diagram (**C**) and community heatmap (**D**) of microbiota composition at the genus level.

The composition of the gut microbiota in mice was analyzed at different taxonomic levels. *Firmicutes* was the main phylum in all three groups (Figure 5A). *Bacteroidota* was the biggest phylum in the DAPT group and *Firmicutes* was the biggest phylum in the MC group. *Proteobacteria* levels were significantly higher in the MC group (vs. NC group). In addition to *Firmicutes* and *Bacteroidota*, *Patescibacteria* and *Actinobacteriota* were also abundant in the NC group. However, in the MC group, the two bacteria were virtually completely absent (Figure 5A). After DAPT treatment, the AkTM-sensitized mice showed a substantial increase in *Bacteroidota* and a significant decrease in *Proteobacteria*. Figure 5B shows the genus-level distribution of the gut microbiota. Gut microbial composition differed greatly between the NC group and the other two groups. Meanwhile, the relative abundance of each bacterium varied greatly between the MC and DAPT group. The gut microbiota heatmap was also clustered into three distinct groups based on similarities and differences in community composition (Figure 5D). A Circos diagram of the gut microbiota (Figure 5C) showed the dominant bacteria at the genus level in each group.

**Figure 6:**
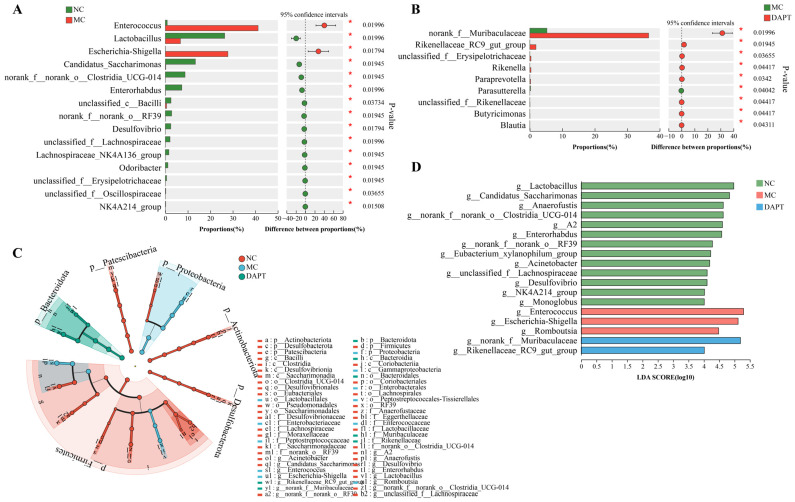
Identification of gut microbiota with significantly differential abundances in different groups (*n* = 5). Bar plot of Wilcoxon rank-sum test on genus level of (**A**) NC vs. MC and (**B**) DAPT vs. MC, * *p* < 0.05; (**C**) taxonomic cladogram shows the microbiota with significantly differential abundances in each group from phylum to genus (from inside to outside). (**D**) Histogram of LDA scores with a threshold > 4 and *p* < 0.05.

The Wilcoxon rank-sum test was performed to identify the differential gut bacteria at the genus level across different groups. Significantly differential abundances of gut microbiota were observed between NC vs. MC groups, and DAPT vs. MC groups (Figure 6A,B). LEfSe was also used to identify microbial taxa that were substantially abundant in different groups of mice. The Cladogram showed the differentially enriched taxa from the phylum to the genus level (Figure 6C). LDA with a score greater than four and a *p* value less than 0.05 was used to select bacteria with significantly differential abundances (Figure 6D). At the genus level, *Enterococcus*, *Escherichia-Shigella*, and *Romboutsia* bacteria were significantly enriched in the MC group, indicating that these bacteria may represent potential gut microbiota signatures of the AkTM allergy. *Muribaculaceae* and *Rikenellaceae_RC9_gut_group* increased significantly after DAPT treatment compared to the other groups. *Lactobacillus*, *Candidatus_Saccharimonas*, *Clostridia_UCG-014*, *Enterorhabdus*, and *Lachnospiraceae* were highly enriched in the NC group (Figure 6D, *p* < 0.05). All the results suggested that these bacteria contributed to the change in gut microbiota composition and community structure in the three groups of mice.

### 3.9. Functional Prediction Analysis of Gut Microbiota

**Figure 7:**
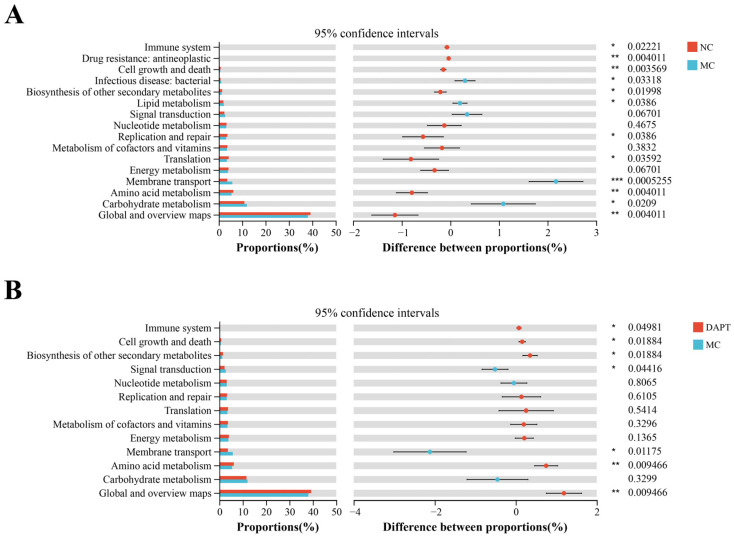
Functional prediction analysis of gut microbiota on KEGG Pathway Level 2 (*n* = 5). Student’s *t*-test of differential functional abundance between (**A**) NC and MC groups; (**B**) DAPT and MC groups. * *p* < 0.05, ** *p* < 0.01 and *** *p* < 0.001.

The functional profiles of gut microbiota in the three groups were predicted using the PICRUSt2 software. In addition, a Student’s *t*-test was performed to distinguish the abundance of functional pathways between groups of interest. On the KEGG pathway level 2, the NC group significantly up-regulated eight pathways including global and overview maps, amino acid metabolism, replication and repair, the biosynthesis of other secondary metabolites, cell growth and death, drug resistance, and the immune system (Figure 7A, *p* < 0.05, vs. MC group), while the MC group up-regulated four pathways including carbohydrate metabolism, membrane transport, lipid metabolism, and infectious disease-bacterial (*p* < 0.05, vs. NC group). Five KEGG pathways on level 2, including the immune system, biosynthesis of other secondary metabolites, cell growth and death, and amino acid metabolism, were much higher in the DAPT group (Figure 7B, *p* < 0.05, vs. MC group). The results indicated that AkTM altered the functional features of gut microbiota in the allergic mice, whereas DAPT treatment could partially restore these functional features through modulating gut microbiota composition and community structure.

### 3.10. Correlation Analysis between Gut Microbiota and SCFAs Concentrations

**Figure 8:**
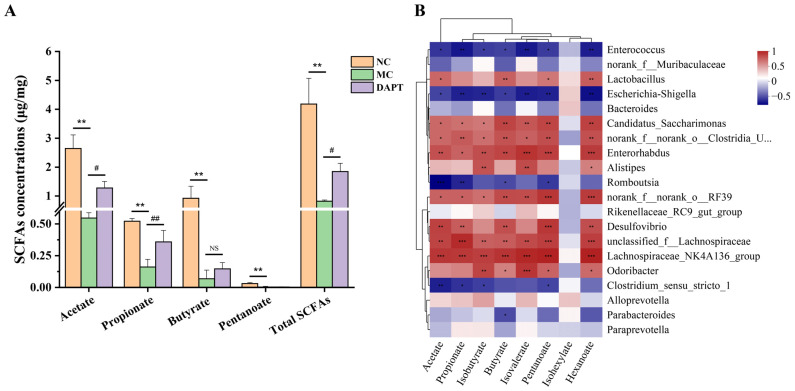
Analysis of SCFA levels and their correlation with the gut microbiota in mice. (**A**) Concentrations of SCFAs detected by GC-MS (*n* = 3). # *p* < 0.05, ## *p* < 0.01, vs. MC group; ** *p* < 0.01, vs. NC group. (**B**) Spearman correlation heatmap between the top 20 bacteria with significantly differential abundances and SCFA concentrations. * *p* < 0.05, ** *p* < 0.01, *** *p* < 0.001, and NS means no significant differences.

Concentrations of SCFAs were evaluated in the colon contents of mice using GC-MS. There were significant differences in SCFA concentrations among different groups. Concentrations of acetate, propionate, isobutyrate, butyrate, isovalerate, pentanoate, hexanoate, and the total SCFAs decreased significantly in the MC group (Figure 8A, *p* < 0.05 or 0.01, vs. NC group). Acetate, propionate, and total SCFAs levels in the DAPT group were higher than those in the MC group (*p* < 0.05 or 0.01), respectively. Moreover, a correlation analysis was performed between SCFA concentrations and differential microbiota features from the three groups. *Candidatus_Saccharimonas*, *Lactobacillus*, *Clostridia_UCG-014*, *Enterorhabdus*, *norank_f_norank_o_RF39*, and *Lachnospiraceae_NK4A136_group* were found to be positively correlated with acetate, propionate, butyrate, pentanoate, and hexanoate (*p* < 0.05). However, *Enterococcus*, *Escherichia-Shigella*, and *Romboutsia* were significantly negatively correlated with these SCFAs (Figure 8B). The corresponding R value of the Spearman correlation is shown in Table S2.

### 3.11. Correlation Analysis between Gut Microbiota and Allergic Indices

To investigate the relationship between gut microbiota and the AkTM allergy, a Spearman correlation analysis was performed between the abundance of bacteria and the levels of allergic indices in different groups. The results showed that *Enterococcus*, *Escherichia-Shigella*, and *Romboutsia* had a significantly positive correlation with AkTM-sIgE (*p* < 0.05), whereas *Lactobacillus*, *Candidatus_Saccharimonas*, *Clostridia_UCG-014*, and *Enterorhabdus* showed a significantly negative correlation with AkTM-sIgE and sIgG1 (Figure S3). In addition, *Candidatus_Saccharimonas* and *Enterorhabdus* presented a significantly negative correlation with serum histamine. mMCP-1 was shown to be positively correlated with *Enterococcus* and *Escherichia-Shigella* (*p* < 0.05) and negatively correlated with *Enterorhabdus* and *Alistipes* (*p* < 0.05). The corresponding R value of Spearman correlation is shown in Table S3. All the results indicated that these bacteria might be associated with the food allergy caused by AkTM.

## 4. Discussion

AkTM has been proven to be a major allergen and capable of inducing IgE-mediated food allergy in mouse models, as our previous work described [2]. In the present study, we used the same BALB/c mouse model with minor modifications to explore the potential mechanisms of AkTM allergy. Increasing evidence shows that Notch signaling appears to play a key role in IgE-mediated food allergy [10,11,15,33]. The Notch signaling pathway is highly conserved and can modulate cell fate decisions and differentiation in lymphocytes and other cells [7,34]. In this study, DAPT, as a γ-secretase inhibitor that can block the Notch signaling pathway [11,15], was used to explore the role of Notch signaling in AkTM-sensitized mice. Notch signaling has been involved in the development of the Th2 immune response and plays a critical role in Th2 cell differentiation [7,13,14]. Food allergies are mostly IgE-mediated immune reactions, with the imbalance of Th1/Th2 responses favoring Th2 polarization [35]. Producing a high level of IgE is a feature of the IgE-mediated Th2 immune response, which is accompanied by the high secretion of Th2 cytokines such as IL-4 and IL-13. In this study, the sensitized mice showed higher levels of AkTM-specific IgE and specific IgG1, enhanced production of IL-4, and lower levels of IFN-γ (Figure 1), suggesting that AkTM-sensitized mice had a Th2-dominant immune response. DAPT treatment decreased specific IgE and IL-4 levels while increasing Th1 cytokine IFN-γ production (Figure 1), demonstrating that inhibiting Notch signaling by DAPT could limit the IgE antibody response, diminish Th2 polarization, and promote the Th1 response. As a result, the findings illustrated that Notch signaling played a critical role in the Th2 immune response in AkTM-sensitized mice. Because IL-4 is primarily responsible for B-cell activation and IgE class transition during the sensitization phase [6], the reduction in IgE levels following DAPT treatment might be due to a decrease in IL-4 levels. Kang et al. reported that the administration of GSI to ovalbumin-sensitized mice could lead to an increase in Th1 cytokines and a decrease in Th2 cytokine production [15], which was in line with our findings. Jiang et al. found that blunting Notch signaling in ovalbumin-allergic mice led to increased levels of IFN-γ and decreased levels of Th2 cytokines [10], and these results were consistent with ours. 

Furthermore, DAPT treatment reduced serum levels of histamine and mMCP-1, and it alleviated anaphylactic symptoms in AkTM-allergic mice (Figure 1), indicating that blocking Notch signaling might suppress mast cell degranulation, thereby alleviating allergic symptoms. Honjo et al. reported that inhibiting Notch signaling could suppress food antigen-induced MMC hyperplasia, resulting in the relief of symptoms related to food allergy [36]. Similarly, Jiang et al. reported that DAPT treatment inhibited mast cell degranulation and cytokine production both in vitro and in vivo [9]. Moreover, the histopathological examination of jejunum tissues showed that DAPT treatment could alleviate damage to the intestinal mucosa and reduce the infiltration of inflammatory cells in allergic mice (Figure 3A). The results of toluidine blue staining indicated that DAPT treatment led to decreased numbers of mast cells and suppressed mast cell degranulation in mice (Figure 3B). Together, these results demonstrated that the inhibition of Notch signaling by DAPT could suppress mast cell degranulation and alleviate AkTM-induced allergic symptoms.

**Figure 9:**
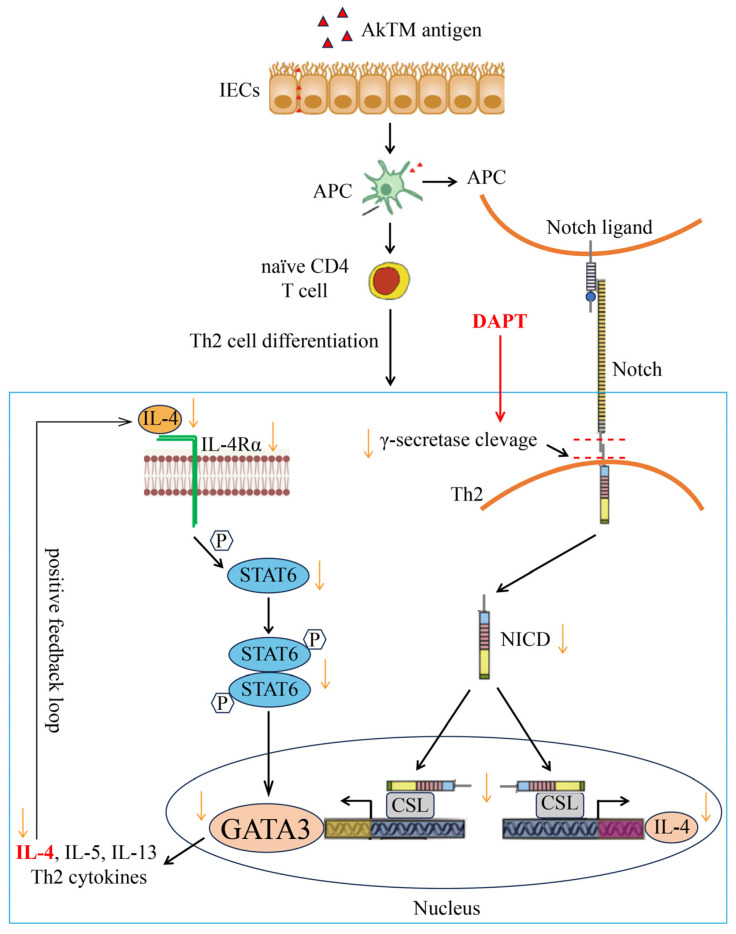
Mechanism diagram of AkTM-induced Th2 cell differentiation in the AkTM-sensitized mice and the effect of DAPT treatment on Th2 differentiation.

To explore the potential sensitization mechanism and the effect of Notch signaling on Th cell differentiation in AkTM-sensitized mice, we examined the expression of some key transcription factors. Hes-1 is the downstream target gene of Notch signaling [37], which was dramatically up-regulated in the MC group (Figure 2), indicating that Notch signaling was active in the AkTM-sensitized mice. DAPT treatment led to significantly decreased Hes-1 expression, suggesting that Notch signaling was effectively inhibited by DAPT. Gata3 and T-bet are critical transcription factors regulating the differentiation of Th2 and Th1 cell lineages, respectively [6,29,30]. Our results revealed that Gata3 expression was dramatically increased in the MC group, whereas it was significantly decreased after DAPT treatment (Figure 2). Meanwhile, the expression of T-bet was reduced in the MC group, whereas it was increased after DAPT treatment (Figure 2). Furthermore, the mRNA levels of Th2 cytokine IL-4 and Th1 cytokine IFN-γ had similar changing trends to Gata3 and T-bet, respectively (Figure 2A). Based on these findings, a conclusion can be drawn that a Th2-dominant immune response occurred in the AkTM-sensitized mice, and that inhibiting Notch signaling could reverse Th1/Th2 imbalance through suppressing the Th2 response and promoting the Th1 response. In agreement with our findings, Jang et al. found that inhibiting Notch signaling could improve Th1/Th2 balance in OVA-sensitized mice [10]. Many studies have demonstrated that Notch directs Th2 cell differentiation by inducing Gata3 and modulating IL-4 gene transcription [7,8]. Moreover, the induction of IL-4 by Notch depends on GATA3 activity [13]. IL-4 can enhance the expression of Gata3 for Th2 differentiation through IL-4R/STAT6 signaling, which is the canonical differentiation pathway of Th2 cells [6]. We found that the expression level of IL-4Rα was significantly increased in the MC group, together with the expression of IL-4 and Gata3, indicating that a canonical Th2 cell differentiation pathway occurred in the AkTM-sensitized mice. It is noteworthy that DAPT treatment effectively reduced IL-4Rα expression (Figure 2). Based on the fact that DAPT treatment reduced the expression of Gata3 and IL-4 in this study, we infer that Notch signaling can directly regulate the expression of the Gata3 gene in Th2 differentiation. Inhibiting Notch signaling decreased the expression of Gata3, thereby reducing the expression of IL-4. Consequently, the positive feedback loop to maintain Th2 cell differentiation was weakened, leading to a decrease in IL-4Rα expression [6]. Kang et al. reported that blocking Notch signaling with GSI could reduce allergic pulmonary inflammation in OVA-sensitized mice by modulating Th1 and Th2 responses [15]. They found that the short-term GSI exposure of BAL cells from allergic mice induced a conversion from Th2 to Th1 cells, concomitant with decreased levels of GATA3 and IL-4, and increased T-bet and IFN-γ expression. This result was consistent with our findings. In short, in our study, the inhibition of Notch signaling by DAPT suppressed Th2 differentiation through down-regulating GATA3 and IL-4 (Figure 9) and promoted Th1 differentiation through up-regulating T-bet and IFN-γ in AkTM-sensitized mice.

Increasing evidence suggests that the dysbiosis of gut microbiota is related to the development of food allergy [38,39,40]. The gut microbiota is crucial in the maintenance of the intestinal immune system. In this study, we used 16S rRNA sequencing to explore the changes in gut microbiota composition and community structure in different groups of mice. We found that AkTM completely reshaped the gut microbiota of mice, reducing microbial diversity and richness. Gu et al. also found that peanut-allergic mice had decreased α-diversity and altered gut microbiota [41]. Chen et al. reported that children with food allergies had a low diversity of total gut microbiota [42]. The enhanced alpha-diversity indices and the separated PCoA and NMDS plots all showed that DAPT treatment alleviated the AkTM-induced reduction in the diversity of gut microbiota and altered the overall microbial structure. The MC group showed higher levels of *Proteobacteria* (Figure 5), and a significant increase in the abundance of *Proteobacteria* is considered to be a potential microbiota signature associated with food allergy [43]. DAPT treatment significantly reduced the level of *Proteobacteria*, suggesting that DAPT might alleviate AkTM-induced food allergy via changing the gut microbial composition.

We further analyzed the bacteria that had significant differences in abundance among the three groups. *Enterococcus* and *Escherichia-Shigella* were more abundant in AkTM-allergic mice, whereas *Lactobacillus*, *Candidatus_Saccharimonas*, *Clostridia_UCG-014*, *Enterorhabdus*, and *Lachnospiraceae* were more abundant in the NC group (Figure 6). *Enterococcus* is a bacterium that causes inflammation by increasing intestinal permeability and impairing the epithelial barrier, resulting in a severe inflammatory response [44]. *Escherichia-Shigella*, which belongs to *Enterobacteriaceae* family, is also an inflammation-inducing bacterium, and its overgrowth might release lots of lipopolysaccharides, which is associated with mucosal inflammation [45]. The two inflammation-inducing bacteria were also detected in the gut and oral microbiota of Kawasaki disease patients [46]. Noval et al. found a significant increase in *Enterobacteriales* and *Enterobacteriaceae* in experimental food-allergic mice [43]. Our findings indicated that *Enterococcus* and *Escherichia-Shigella* were positively related to AkTM allergy, as evidenced by the positive correlation with allergic indices including AkTM-sIgE, sIgG1, and mMCP-1 (Figure S3). The increased *Enterococcus* and *Escherichia-Shigella* induced by AkTM, on the other hand, were reduced after DAPT treatment (Figure 5B,C), suggesting that DAPT might alleviate AkTM-induced allergy by restricting the proliferation of these harmful bacteria. Gu et al. found a lower expression of *Lachnospiraceae*, *Alistipes*, and *Lachnospiraceae_NK4A136_group* in the peanut-allergic mice [41], which agreed with our findings. *Lachnospiraceae* can produce SCFAs, such as butyric acid, which can relieve allergic symptoms and reduce serum IgE in peanut-allergic animals [47]. *Lactobacillus* and *Candidatus_Saccharimonas* are beneficial bacteria that are shown to be elevated in an egg white peptide-treated colitis model, indicating their potential immunomodulatory effect in the gut [48]. Studies showed that colonization with *Clostridia* in germ-free mice could reduce peanut sensitization [49], and *Clostridia* species effectively relieved symptoms in an intestinal allergy model [50]. It was reported that *Enterorhabdus* was negatively correlated with proinflammatory cytokine levels and that it might play an active role in intestinal homeostasis [51]. Likewise, we found a negative correlation between *Enterorhabdus* and certain allergic indices (Figure S3). All of the previously indicated potentially beneficial bacteria were down-regulated in the AkTM-allergic mice, whereas DAPT treatment could partially restore these gut bacterial compositions (Figure 5). A lower expression of *Rikenellaceae* was observed in the gut of peanut-sensitized mice [41], which was in line with our findings of decreased abundance of *Rikenellaceae* in AkTM-allergic mice (Figure 6). Therefore, we infer that *Rikenellaceae* may play a crucial role in preventing food allergy [52].

SCFAs have been proven to have an important role in protecting against food allergy and generating immunological tolerance [21,22]. Goldberg et al. found decreased SCFA levels in food-allergic individuals [19], which was consistent with our findings of lower SCFA levels in AkTM-allergic mice. DAPT treatment could alleviate AkTM-induced decreases in SCFA levels, particularly acetate and propionate (Figure 8A). Most potentially beneficial bacteria, including *Candidatus_Saccharimonas*, *Lactobacillus*, *Clostridia_UCG-014* and *Lachnospiraceae*, were positively correlated with SCFA concentrations (Figure 8B). Propionate treatment in mice could enhance the generation of DC precursors, which inhibits Th2 cells, thereby reducing allergic inflammation [24]. Butyrate, which is mainly produced by Clostridial clusters, has been shown to suppress many inflammatory responses as well as stimulate Treg cell proliferation in the formation of immunological tolerance to food allergens [47,53]. In our research, *Clostridia_UCG-014* was likewise shown to be positively correlated with butyrate. *Lachnospiraceae* is an SCFA-producing bacterium that can decrease proinflammatory cytokine levels while increasing anti-inflammatory cytokine production [54]. Tan et al. reported that treating mice with acetate and butyrate could protect against food allergy by reducing serum IgE levels and anaphylaxis scores [47]. They also found that SCFAs had no effect on food allergy suppression in the absence of gut microbiota. Therefore, it is recommended that gut microbiota and their metabolites should be studied together to evaluate their roles in food allergies. Moreover, we found that several inflammation-causing bacteria, such as *Enterococcus* and *Escherichia-Shigella*, were substantially negatively correlated with SCFA levels in AkTM-allergic mice.

## 5. Conclusions

In this research, we explored the potential sensitization mechanism of an AkTM-sensitized mouse model by inhibiting Notch signaling using a DAPT-γ-secretase inhibitor. AkTM could elicit an IgE-mediated, Th2-dominant immune response with a Th1/Th2 imbalance. In AkTM-sensitized mice, inhibiting Notch signaling could restore Th1/Th2 imbalance, alleviate allergic symptoms, and reduce intestinal inflammation. AkTM altered the composition and overall community structures of the gut microbiota, causing an increase in certain inflammation-inducing bacteria. Moreover, SCFA concentrations were lower in the allergic mice. It is noteworthy that inhibiting Notch signaling could diminish some harmful bacteria induced by AkTM while restoring beneficial bacteria, particularly some SCFA-producing bacteria. Allergic-related indices were shown to be positively correlated with harmful bacteria, implying that lowering the abundance of harmful bacteria might reduce food allergy. In conclusion, our findings indicate that inhibiting Notch signaling may be an effective strategy to improve AkTM-induced food allergy. Future work should investigate the mechanism by which SCFAs modulate AkTM allergy and the relationship between gut microbiota metabolites and food allergies.

## Data Availability

The original contributions presented in the study are included in the article/supplementary material, further inquiries can be directed to the corresponding authors.

## Supplementary Materials

The following supporting information can be downloaded at https://www.mdpi.com/article/10.3390/foods13081144/s1, Table S1: Primer sequences for qPCR; Table S2: R values of the Spearman correlation between the top 20 bacteria with significantly differential abundances and SCFA concentrations; Table S3: R values of the Spearman correlation between the top 10 bacteria with significantly differential abundances and allergic indices in different groups (*n* = 5); Figure S1: Protocol for the AkTM-sensitized and DAPT-treated mice model; Figure S2: The β-diversity analysis of gut microbiota in mice (*n* = 5 for each group). (A) PCoA analysis based on weighted unifrac distances; (B) Hierarchical clustering tree on genus level based on Bray–Curtis distance; Figure S3: Heatmap of Spearman correlation between the top 10 bacteria with significantly differential abundances and allergic indices in different groups (*n* = 5). * *p* < 0.05, ** *p* < 0.01, and *** *p* < 0.001; Figures S4–S9: Western blotting raw images of Gata3, T-bet, Hes-1, IL-4Rα, NF-κB, p-NF-κB, and β-actin.
