# Supplementary material for: Notch Signaling Inhibition Alleviates Allergies Caused by Antarctic Krill Tropomyosin through Improving Th1/Th2 Imbalance and Modulating Gut Microbiota
Source: Foods. 2024 Apr 9;13(8):1144. doi: 10.3390/foods13081144 (PMC11048830; doi:10.3390/foods13081144)
Supplement: Supplementary file 1 [file foods-13-01144-s001.zip › foods-2872213-supplementary.pdf]

*Supplementary materials*

**Table S1.** Primer sequences for qPCR.

| Gene name | Forward primer sequences (5'-3') | Reverse primer sequences (5'-3') |
|---|---|---|
| IFN-γ | GGTCAACAACCCACAGGTCCA | GCGACTCCTTTTCCGCTTCCT |
| IL-4 | CGGAGATGGATGTGCCAAACG | TGGAAGCCCTACAGACGAGC |
| Hes-1 | ATGTGGAGACCGTGGCGGAA | CGTCAGAAGAGAGAGGTGGGC |
| Gata-3 | TATCCGCCCTATGTGCCCGA | AGGTTGCCCCGCAGTTCAC |
| T-bet | TCAACTGCTTGGGGGAGACC | GGGGTAGAAACGGCTGGGAAC |
| NF-κB | GGTGTGGAGACATCCTTCCGC | CCTTCCTGCCCATAACCGTGG |
| GAPDH | AGGAGCGAGACCCCACTAAC | CGGAGATGATGACCCTTTTGGCT |

**Table S2.** R values of the Spearman Correlation between the top 20 bacteria with significantly differential abundances and SCFAs concentrations.

| | Acetate | Propionate | Isobutyrate | Butyrate | Isovalerate | Pentanoate | Isohexylate | Hexanoate |
|---|---|---|---|---|---|---|---|---|
| g__Enterococcus | -0.6176 | -0.7055 | -0.5912 | -0.5736 | -0.6967 | -0.5956 | -0.1824 | -0.6909 |
| g__norank_f__Muribaculaceae | -0.4637 | -0.2703 | 0.0813 | -0.4813 | 0.1165 | -0.3934 | -0.0418 | -0.3542 |
| g__Lactobacillus | 0.6352 | 0.4945 | 0.3451 | 0.6615 | 0.4769 | 0.5736 | 0.1956 | 0.6733 |
| g__Escherichia-Shigella | -0.5667 | -0.6800 | -0.6712 | -0.5867 | -0.6912 | -0.6756 | 0.2489 | -0.7375 |
| g__Bacteroides | -0.2088 | -0.2835 | 0.0725 | -0.4154 | 0.0198 | -0.3451 | 0.2747 | -0.4114 |
| g__Candidatus_Saccharimonas | 0.6336 | 0.5982 | 0.5673 | 0.7660 | 0.6954 | 0.7572 | -0.0596 | 0.7713 |
| g__norank_f__norank_o__Clostridia_ UCG-014 | 0.6336 | 0.6821 | 0.6071 | 0.7086 | 0.6601 | 0.7263 | -0.2671 | 0.7204 |
| g__Enterorhabdus | 0.7187 | 0.6527 | 0.7275 | 0.7582 | 0.8286 | 0.7846 | 0.0549 | 0.8031 |
| g__Alistipes | 0.3319 | 0.3055 | 0.7099 | 0.3890 | 0.7407 | 0.5165 | -0.1297 | 0.5413 |
| g__Romboutsia | -0.7890 | -0.6676 | -0.5013 | -0.5462 | -0.4383 | -0.6002 | -0.0787 | -0.4523 |
| g__norank_f__norank_o__RF39 | 0.6512 | 0.6556 | 0.6027 | 0.7395 | 0.7351 | 0.8190 | -0.1965 | 0.8022 |
| g__Rikenellaceae_RC9_gut_group | -0.0155 | 0.0817 | 0.2406 | -0.0993 | 0.3068 | 0.0949 | -0.2009 | -0.0751 |
| g__Desulfovibrio | 0.7768 | 0.6736 | 0.4905 | 0.7252 | 0.5093 | 0.8144 | -0.1948 | 0.7260 |
| g__unclassified_f__Lachnospiraceae | 0.7613 | 0.8625 | 0.6645 | 0.6755 | 0.7437 | 0.7833 | -0.0506 | 0.7885 |
| g__Lachnospiraceae_NK4A136_group | 0.8190 | 0.8057 | 0.7837 | 0.8278 | 0.8720 | 0.8896 | 0.1038 | 0.8950 |
| g__Odoribacter | 0.4884 | 0.5282 | 0.7647 | 0.5414 | 0.8221 | 0.6475 | -0.2387 | 0.6261 |
| g__Clostridium_sensu_stricto_1 | -0.6823 | -0.6223 | -0.5800 | -0.5178 | -0.5000 | -0.5623 | 0.0156 | -0.4272 |
| g__Alloprevotella | 0.2133 | 0.2778 | 0.4134 | -0.0178 | 0.3289 | 0.0356 | 0.2778 | -0.0845 |
| g__Parabacteroides | -0.2356 | -0.1467 | -0.0667 | -0.5423 | -0.0689 | -0.4378 | 0.1045 | -0.5028 |
| g__Paraprevotella | -0.1760 | 0.1432 | 0.1479 | -0.2511 | 0.1009 | -0.0962 | -0.1103 | -0.1504 |

**Table S3.** R values of the Spearman Correlation between the top 10 bacteria with significantly differential abundances and allergic indices in different groups ($n = 5$).

| | sIgE | sIgG1 | His | mMCP1 |
|---|---|---|---|---|
| g__Enterococcus | 0.6396 | 0.5780 | 0.3275 | 0.5516 |
| g__norank_f__Muribaculaceae | 0.1736 | 0.5297 | 0.1516 | -0.2571 |
| g__Lactobacillus | -0.6571 | -0.8418 | -0.3714 | -0.3538 |
| g__Escherichia-Shigella | 0.7245 | 0.3822 | 0.4711 | 0.7089 |
| g__Bacteroides | 0.1385 | 0.3538 | 0.0681 | -0.2615 |
| g__Candidatus_Saccharimonas | -0.7395 | -0.7660 | -0.5453 | -0.5099 |
| g__norank_f__norank_o__Clostridia_UCG-014 | -0.7572 | -0.7174 | -0.4305 | -0.5320 |
| g__Enterorhabdus | -0.8110 | -0.6879 | -0.6747 | -0.6352 |

| | | | | |
|---|---|---|---|---|
| g__Alistipes | -0.4286 | -0.0242 | -0.3143 | -0.5780 |
| g__Romboutsia | 0.5777 | 0.6316 | 0.3259 | 0.3664 |

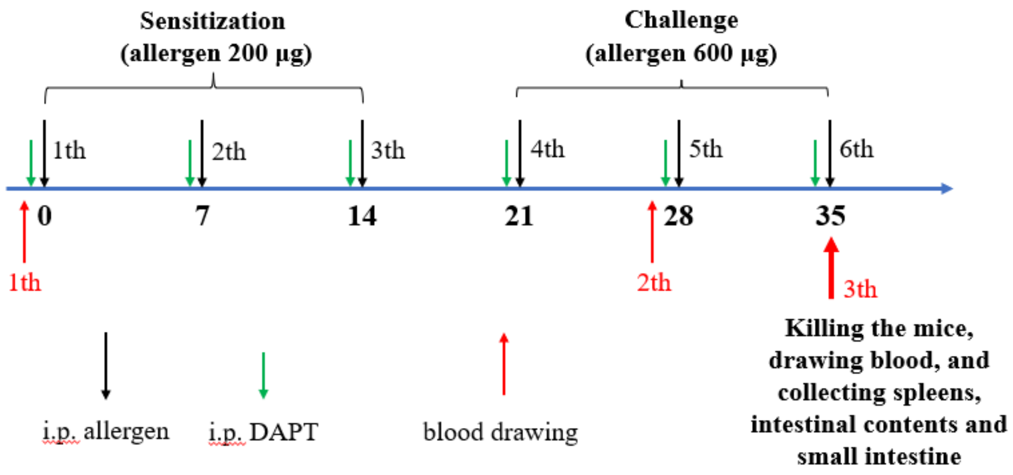


**Figure S1.** Protocol for the AkTM-sensitized and DAPT-treated mice model.

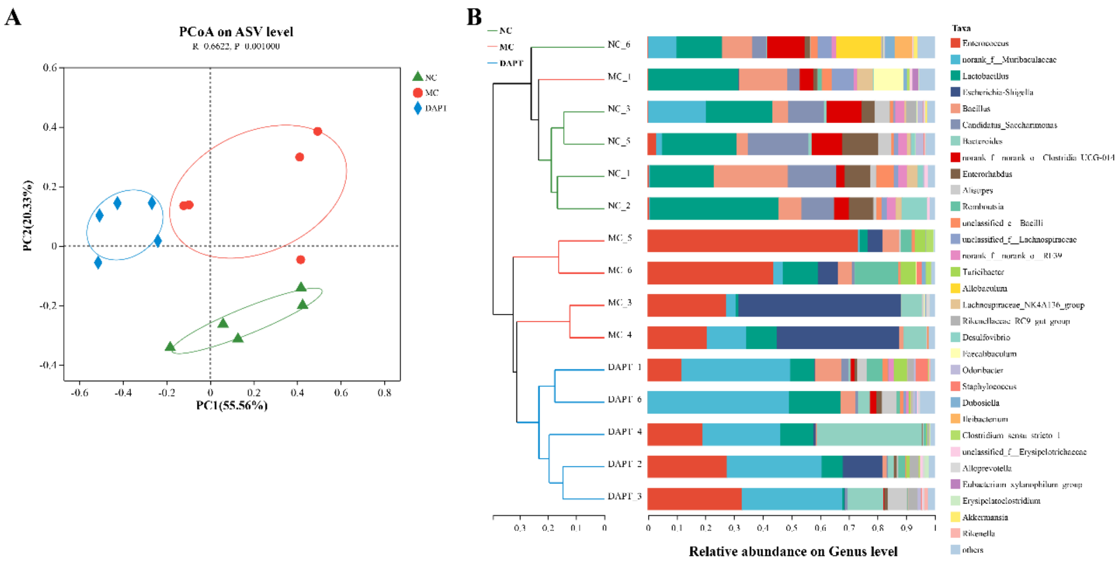


**Figure S2.** The β-diversity analysis of gut microbiota in mice (n = 5 for each group). (A) PCoA analysis based on weighted unifrac distances; (B) Hierarchical clustering tree on genus level based on Bray-curtis distance.

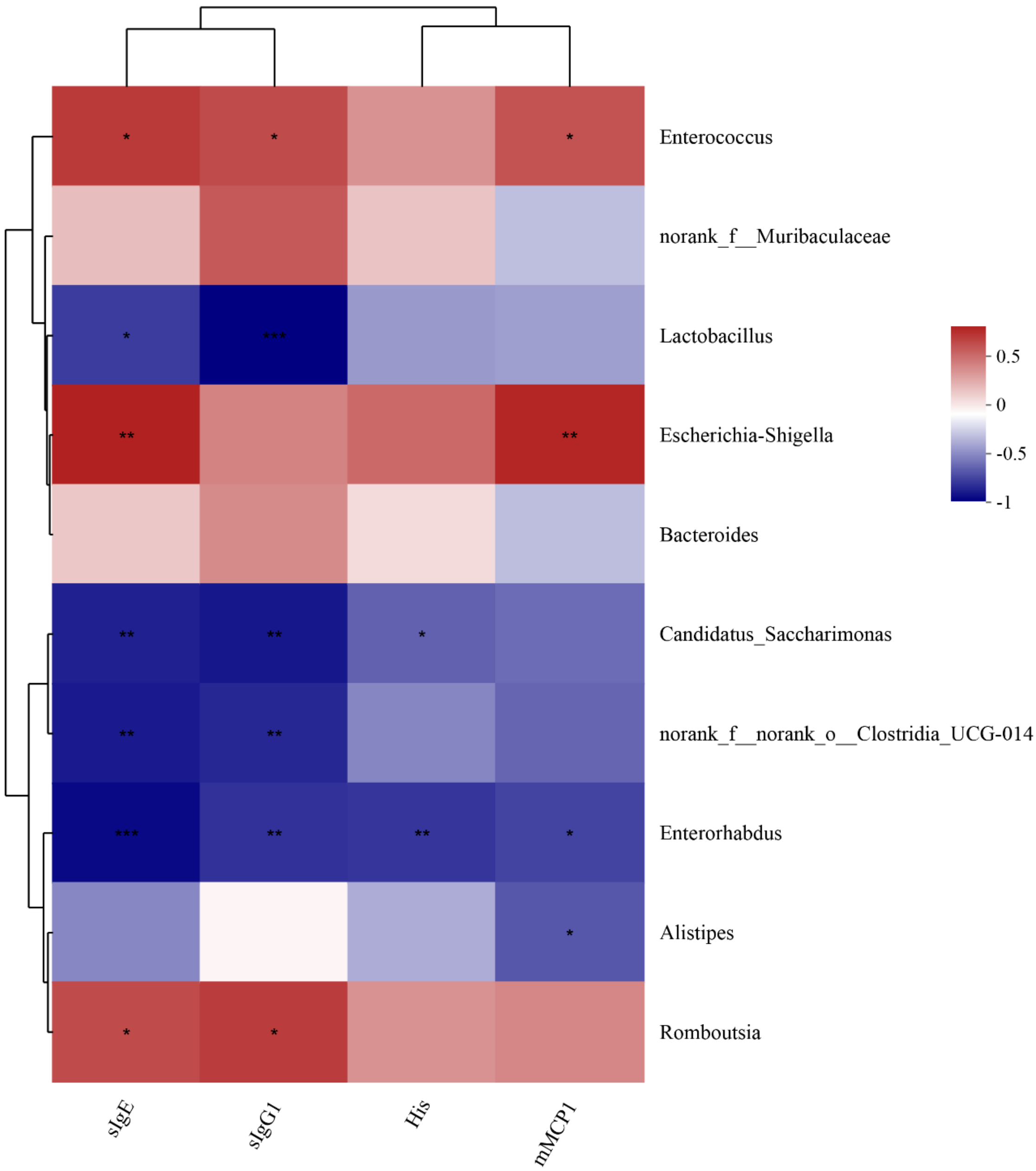


**Figure S3.** Heatmap of spearman correlation between the top 10 bacteria with significantly differential abundances and allergic indices in different groups ($n$ = 5). * $p < 0.05$, ** $p < 0.01$, and *** $p < 0.001$.

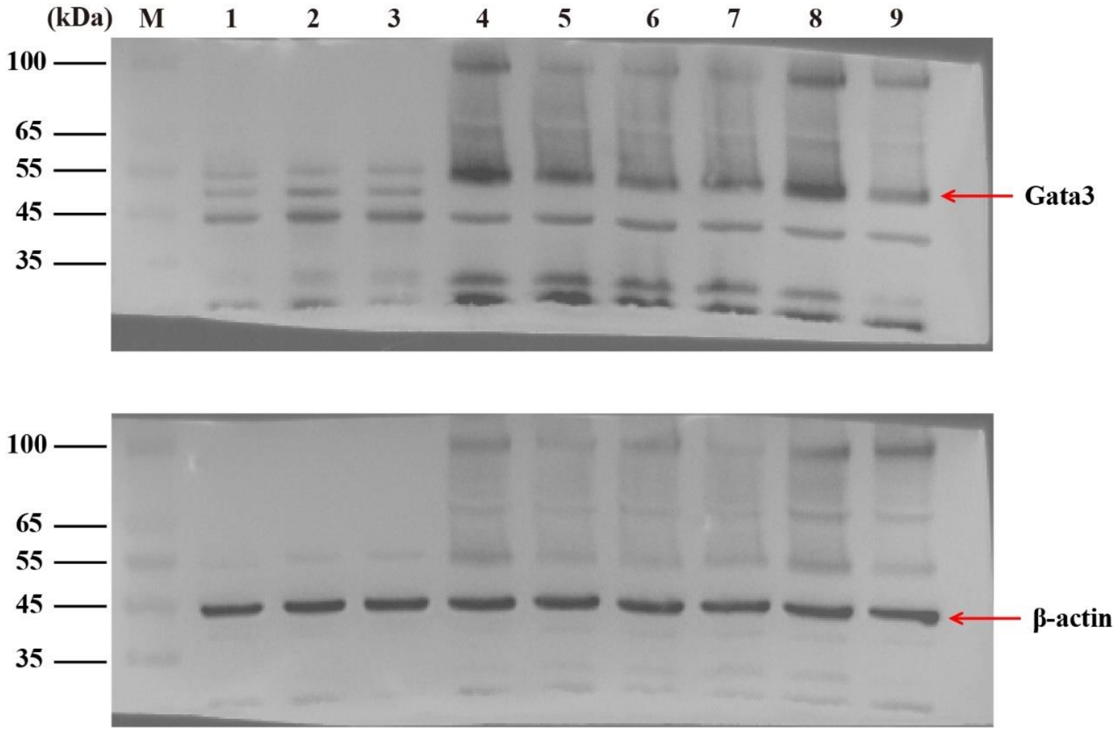


**Figure S4.** Western blotting raw images of Gata3 and β-actin. Three biological samples per group of mice. M, protein markers; 1, 2, and 3, NC group; 4, 5, and 6, MC group; 7, 8, and 9, DAPT group. Gata3 bands of lanes 3, 4, 7, and β-actin bands of lanes 3, 4, 7, were used in Figure 2C in the manuscript.

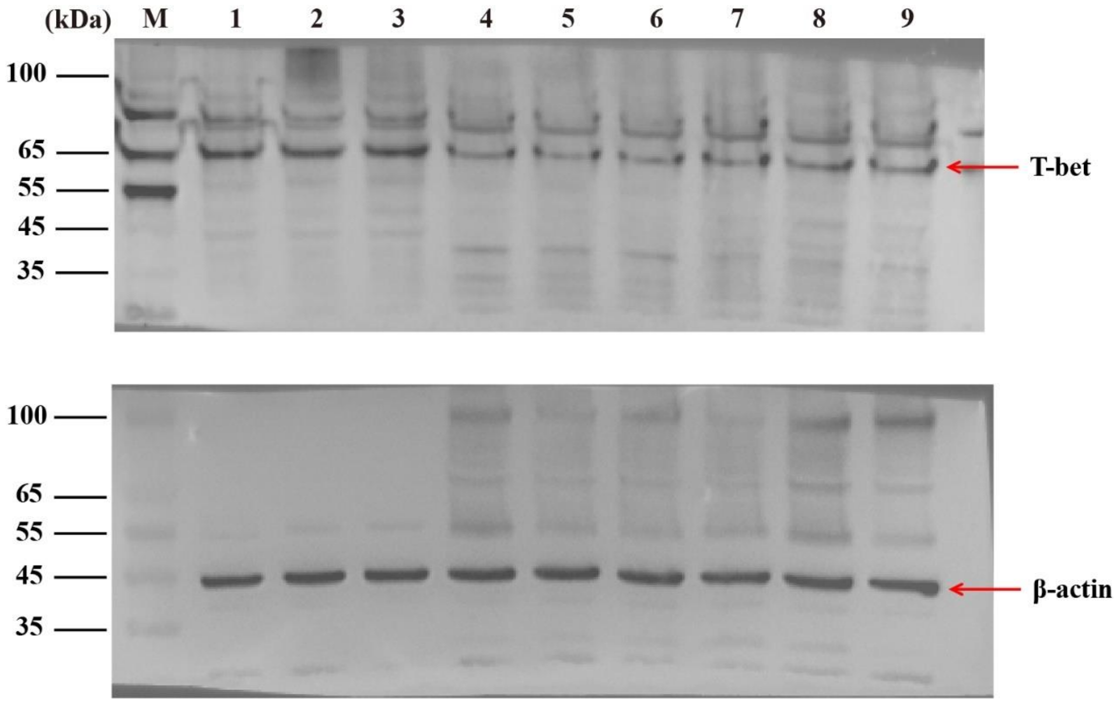


**Figure S5.** Western blotting raw images of T-bet and β-actin. Three biological samples per group of mice. M, protein markers; 1, 2, and 3, NC group; 4, 5, and 6, MC group; 7, 8, and 9, DAPT group. T-bet bands of lanes 3, 4, and 8 were used in Figure 2C in the manuscript.

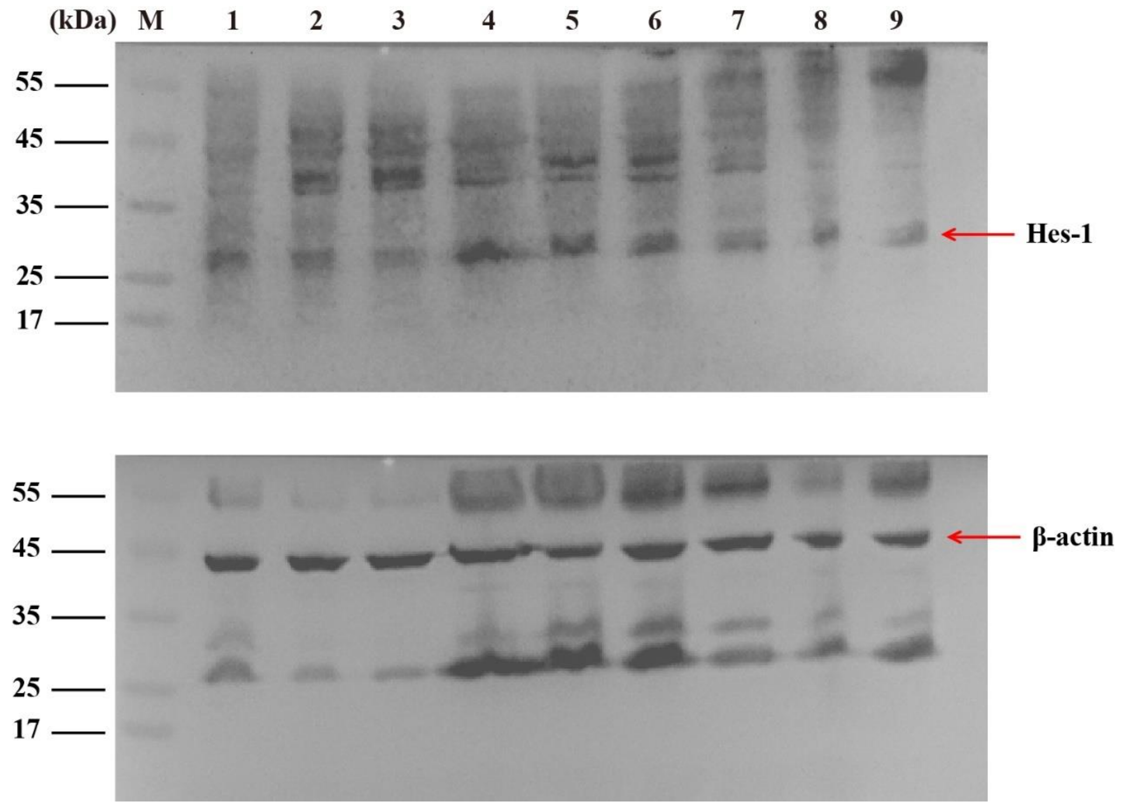


**Figure S6.** Western blotting raw images of Hes-1 and β-actin. Three biological samples per group of mice. M, protein markers; 1, 2, and 3, NC group; 4, 5, and 6, MC group; 7, 8, and 9, DAPT group. Hes-1 bands of lanes 3, 4, and 7 were used in Figure 2C in the manuscript.

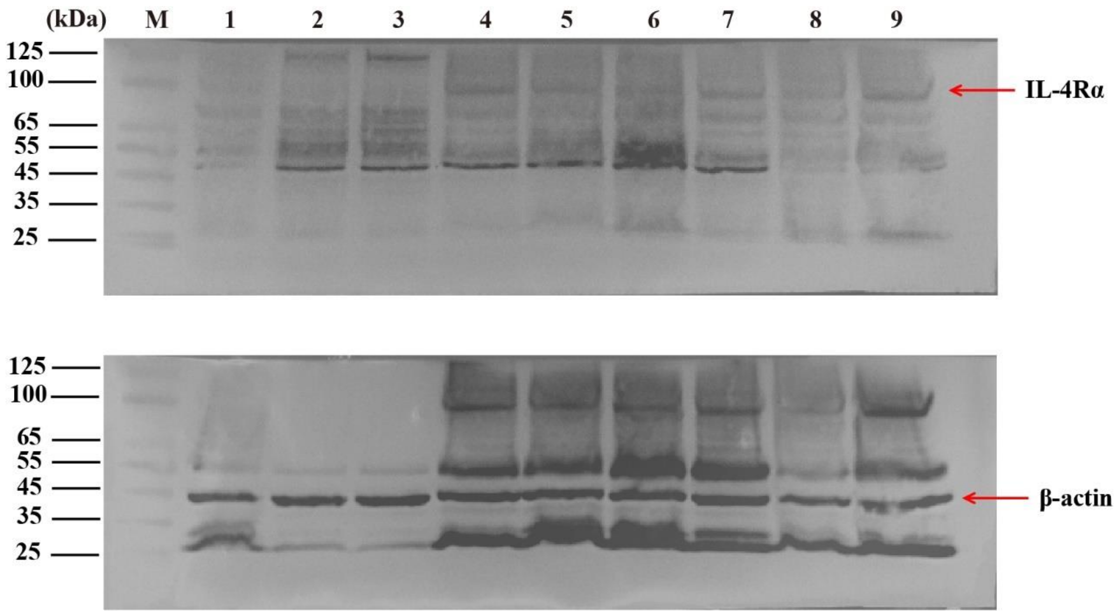


**Figure S7.** Western blotting raw images of IL-4Rα and β-actin. Three biological samples per group of mice. M, protein markers; 1, 2, and 3, NC group; 4, 5, and 6, MC group; 7, 8, and 9, DAPT group. IL-4Rα bands of lanes 3, 4, and 7 were used in Figure 2C in the manuscript.

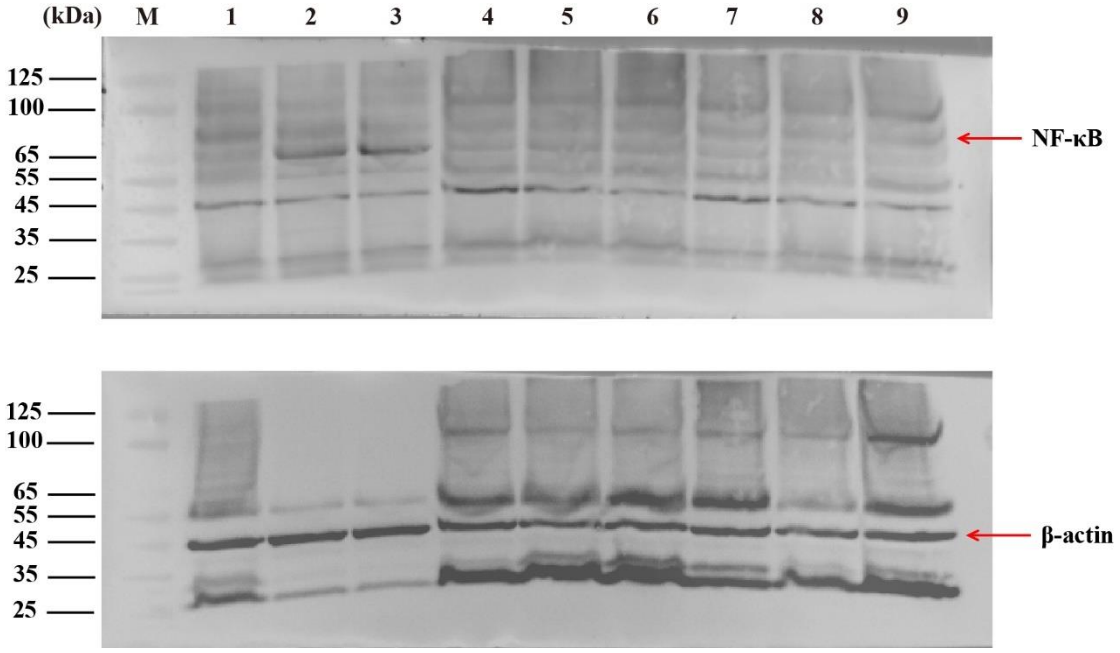


**Figure S8.** Western blotting raw images of NF-κB and β-actin. Three biological samples per group of mice. M, protein markers; 1, 2, and 3, NC group; 4, 5, and 6, MC group; 7, 8, and 9, DAPT group. NF-κB bands of lanes 3, 4, and 7 were used in Figure 2C in the manuscript.

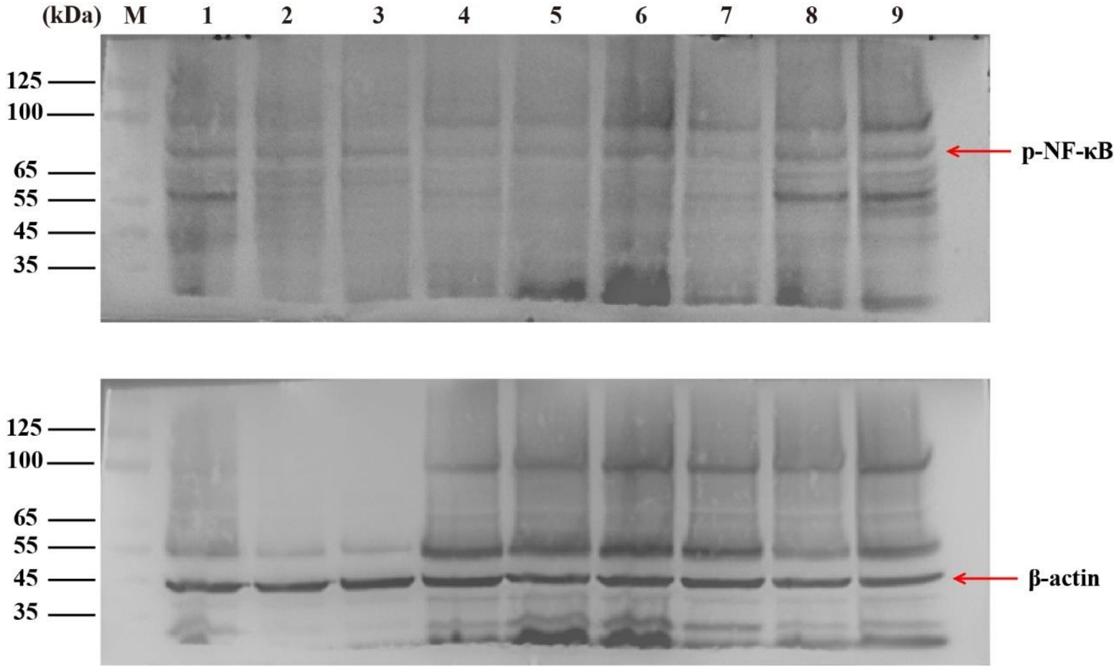


**Figure S9.** Western blotting raw images of p-NF-κB and β-actin. Three biological samples per group of mice. M, protein markers; 1, 2, and 3, NC group; 4, 5, and 6, MC group; 7, 8, and 9, DAPT group. p-NF-κB bands of lanes 3, 6, and 7 were used in Figure 2C in the manuscript.
